# Scalp Metastasis From Renal Cell Carcinoma: A Rare Case With Rapid Progression

**DOI:** 10.7759/cureus.72546

**Published:** 2024-10-28

**Authors:** Mohammed S Alzahrani, Abdulsamad O Alharbi, Abdulaziz Alghamdi, Abdulrahman Mojallid, Mohamed Alghamdi, Abdullrahman Alayyad, Awnallah Alotaibi, Abdulrhman A Alamri, Rehab Fadag, Mohamed Sheta, Abdulrahman S Khalil, Ibrahem F Alassiri, Abdulghani Khogeer, Adel Elatreisy

**Affiliations:** 1 Urology, King Fahd Armed Forces Hospital, Jeddah, SAU; 2 Anatomical Pathologist, Histopathology, King Fahd Armed Forces Hospital, Jeddah, SAU; 3 Oncology, King Fahd Armed Forces Hospital, Jeddah, SAU; 4 Radiology, King Fahd Armed Forces Hospital, Jeddah, SAU; 5 Surgery, Faculty of Medicine, Rabigh, King Abdulaziz University, Jeddah, SAU; 6 Urology, Faculty of Medicine, Al-Azhar University, Cairo, EGY

**Keywords:** nephrectomy, renal cell carcinoma, scalp metastases, skin metastases, surgical excision

## Abstract

Renal cell carcinoma (RCC) is a common renal malignancy that frequently metastasizes, though cutaneous metastasis, particularly to the scalp, is rare and generally indicates a poor prognosis. We report a case of scalp metastasis from RCC in a 69-year-old man who presented with unexplained weight loss and painless hematuria. Imaging revealed a 12-cm renal mass with adrenal, pulmonary, and scalp metastases. The patient’s condition rapidly deteriorated, and he died one month after the presentation. Hematogenous spread through Batson’s plexus might be the primary mechanism of RCC dissemination to the scalp. While targeted therapies have improved the management of metastatic RCC, the prognosis for patients with skin metastases remains poor.

## Introduction

Renal cell carcinoma (RCC) is the most common renal malignancy, accounting for more than 3% of adult solid tumors and consisting of several histological subtypes, with clear cell RCC being the most prevalent [1]. RCC predominantly affects men and has an increasing incidence in older adults, particularly between the fifth and seventh decades of life [1,2].

RCC frequently metastasizes via hematogenous spread, with approximately one-third of patients developing metastases [3,4]. The lungs are the most common site of metastasis, followed by the bones, lymph nodes, liver, brain, and adrenal glands [3]. Metastasis to the skin is rare and generally indicates a poor prognosis [5]. To the best of our knowledge, we report the 27th documented case of RCC metastasizing to the scalp, characterized by rapid progression and ultimately fatal outcomes. A review of previously published cases is also included.

## Case presentation

**Figure 1:**
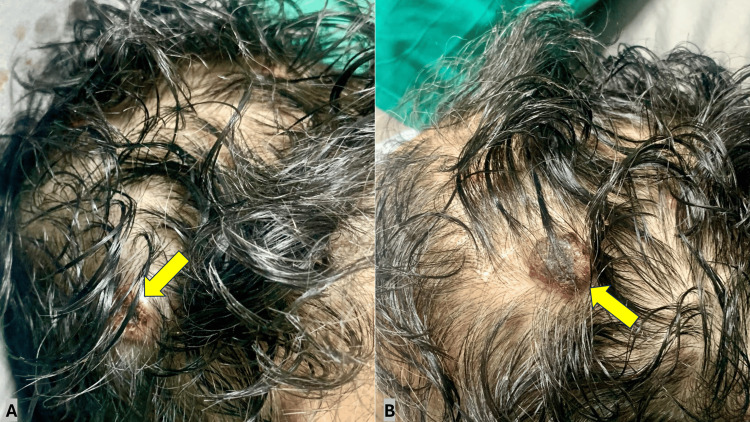
Multiple metastatic scalp lesions originating from renal cell carcinoma (A and B)

A 69-year-old male presented to the clinic with unexplained weight loss for the last few weeks with recurrent attacks of visible painless hematuria. He had a first-degree family history of RCC. Examination showed multiple scalp cutaneous metastasis (Figure 1).

**Figure 2:**
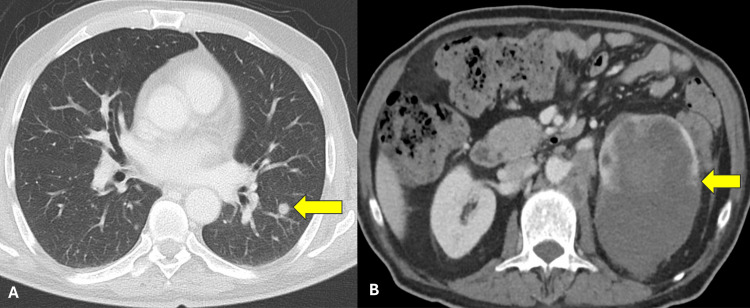
Computed tomography findings A: CT chest with intravenous contrast showing left pulmonary metastases (arrow); B: CT abdomen with intravenous contrast showing a Bosniak category IV left renal cystic mass invading the left adrenal gland (arrow) with multiple retroperitoneal lymphadenopathies

**Figure 3:**
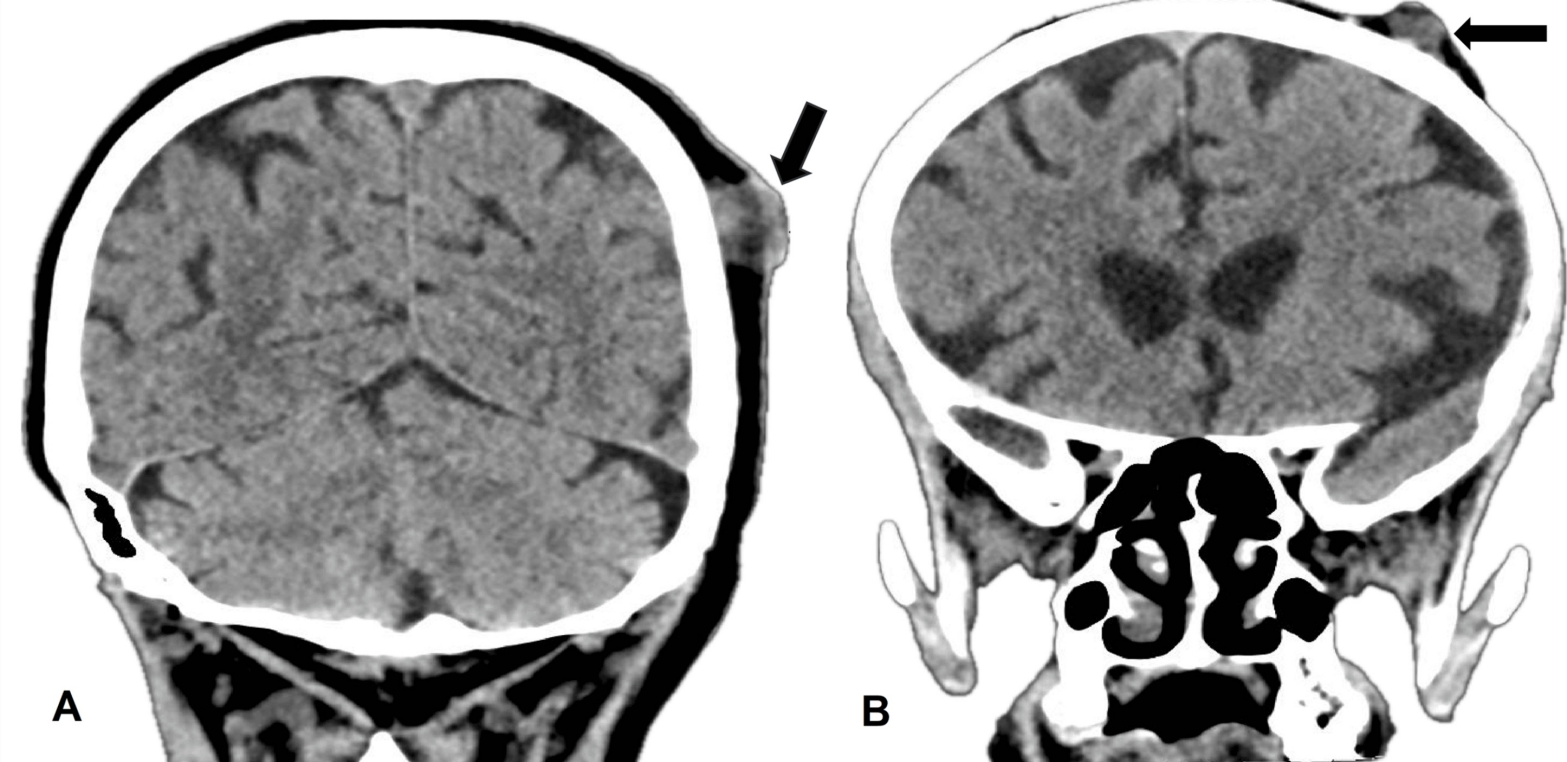
Computed tomography findings A: CT brain showing a metastatic renal cell carcinoma scalp mass in the temporal area (arrow); B: CT brain showing a metastatic renal cell carcinoma scalp mass in the frontal area (arrow)

**Figure 4:**
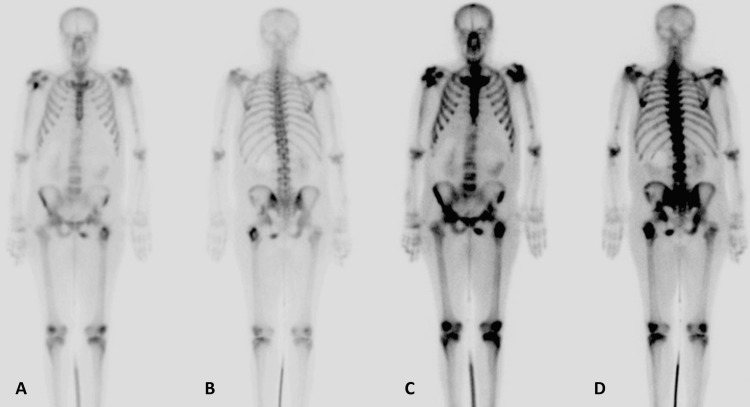
Bone scan showing widespread osseous metastases (A, B, C, D)

Laboratory investigations showed low hemoglobin (84 gm/l), high platelets, neutrophils, and calcium. A computed tomography (CT) scan showed Bosniak category IV, 12 cm left renal cystic mass infiltrating the left adrenal gland with multiple retroperitoneal lymphadenopathies and multiple pulmonary metastases (Figure 2). The CT brain showed multiple cutaneous scalp metastasis (Figure 3), while the bone scan showed generous bone metastasis (Figure 4).

**Figure 5:**
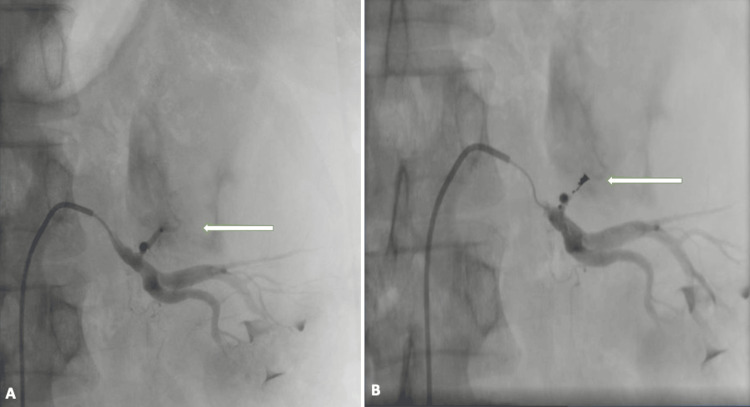
Angiography findings A: left renal angiography showing the feeding artery for the upper pole mass of the left kidney (arrow); B: selective angioembolization of the tumor-feeding artery in the left renal mass showing the coil occluding the artery (arrow)

A few days after his presentation, he was admitted to the emergency department with frank hematuria and a hemoglobin drop to 63 gm/l, where he was transfused with two units of packed RBCs. On admission, he underwent a punch biopsy from the scalp lesions and a CT-guided true-cut renal mass biopsy with selective angioembolization of the tumor-feeding artery (Figure 5).

**Figure 6:**
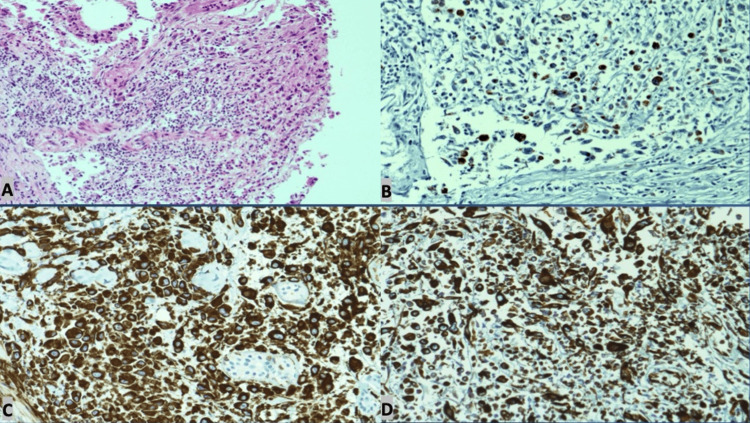
Renal biopsy at 10x magnification A: infiltrative poorly differentiated epithelioid to spindle cells exhibiting pleomorphic, hyperchromatic nuclei with prominent nucleoli and eosinophilic cytoplasm; B: tumor cells positive for EMA; C: tumor cells positive for panCK; D: tumor cells positive for vimentin CT: computed tomography; EMA: epithelial membrane antigen; panCK: pan-cytokeratin

Histopathology from renal mass showed infiltrative, poorly differentiated epithelioid to spindle cells, RCC, and tumor cells were positive for epithelial membrane antigen (EMA), pan-cytokeratin (panCK), and vimentin (Figure 6).

**Figure 7:**
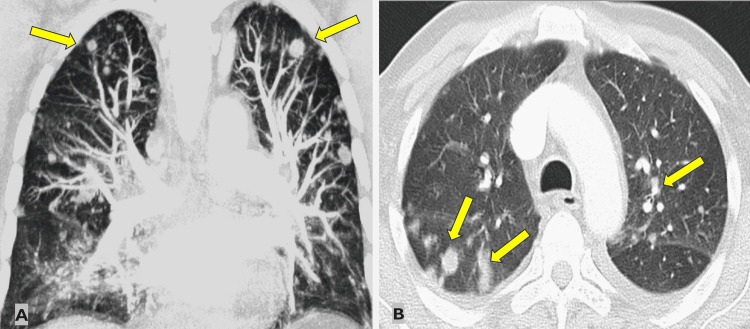
CT chest showing progressive pulmonary metastases (A and B) CT: computed tomography

Scalp lesions showed RCC metastasis. His Tumor Node Metastasis (TNM) staging was T4N1M1, and the International Metastatic RCC Database Consortium (IMDC) score of 5/6 [6] classified him as a poor-risk patient. A multidisciplinary team decided to start systemic treatment. The patient had progressive chest metastasis (Figure 7), and he passed away one month after his first presentation.

## Discussion

Metastatic RCC to the skin is rare, accounting for 1% to 6.5% of all RCC metastases, with the scalp and face being the most commonly affected sites [1]. Our literature review identified 26 cases of RCC metastasis to the scalp. The mean age of the patients was 57.7 years (range: 30-83 years), with a male predominance of 81.5% [1-22]. In 33.3% of cases, scalp metastasis occurred in isolation, while 66.7% of cases involved metastases to other sites. Among these, 14 patients had undergone nephrectomy for RCC prior to presenting with scalp lesions. The mean time between primary RCC treatment and the diagnosis of scalp metastasis was 33 months (range: 0-180 months). In 37% of cases, scalp metastasis was the initial presentation, and five patients were subsequently diagnosed with RCC following nephrectomy. Surgical excision was the most common treatment for scalp metastases (n=16), while some cases were managed with radiotherapy (two cases), chemotherapy/interferon (three cases), or chemoradiotherapy (one case). A summary of all reported cases of metastatic RCC to the scalp from 1977 to 2024 is provided in Table 1 [1-5,7-23].

**Table 1:** Summary of published cases of renal cell carcinoma metastatic to the scalp NR: not recorded

Author, Year	Sex	Age in Years	First Presentation	Treatment to Primary	Location of Other Metastases	Treatment to Scalp Metastases	Time to Skin Metastases (Months)	Survival After Detection of Skin Metastasis (Months)
Livingston et al., 1977 [11]	M	45	Scalp lesion	Nephrectomy	None	Surgical excision	0	NR
Wahner-Roedler et al., 1997 [10]	F	72	Scalp lesion	Nephrectomy	None	Radiotherapy	0	15
Dorairajan, et al, 1999 [7]	M	55	NR	Nephrectomy	Abdomen, brain	Interferon	10	4
	M	55	Scalp lesion	None	Liver, bone	None	0	3
	M	30	Scalp lesion	None	Liver, bones, brain	Surgical excision	0	3
	M	40	Scalp lesion	None	Lung	Interferon	0	8
	M	56	NR	Nephrectomy	Bones	None	19	7
Snow et al., 2001 [12]	F	69	Hematuria	Nephrectomy	Bicep, mediastinum, spine	Surgical excision	72	NR
Eke et al., 2003 [13]	F	42	Scalp lesion, hematuria	Nephrectomy	None	None	0	NR
Estrada-Chavez et al., 2006 [3]	M	80	Incidental cystic renal mass	Nephrectomy	None	None	48	NR
Pan et al., 2006 [23]	M	63	Scalp lesion	Nephrectomy	None	Surgical excision	0	NR
Smyth et al., 2010 [14]	M	67	NR	Nephrectomy	Left ureter, rectus abdominus muscle, pancreas, brain	Surgical excision	120	9
De Paula et al., 2010 [15]	M	61	Incidental renal mass	Nephrectomy was not done due to heart failure decompensation	Lung	Surgical excision	0	Dead from acute respiratory failure
Abbasi et al., 2013 [16]	M	42	Hematuria	Nephrectomy and chemotherapy	None	Surgical excision	1	NR
Anzalone et al., 2013 [17]	M	52	NR	Chemotherapy	None	Surgical excision	30	24
Errami et al., 2016 [5]	M	64	Incidental renal mass	Nephrectomy	Tonsils, cervical lymphadenopathy, bone	Chemotherapy	36	NR
Selvi et al., 2016 [18]	M	51	NR	Nephrectomy	Oral cavity, brain, lung	Surgical excision	36	6
Ferhatoglu et al., 2018 [19]	F	40	NR	Nephrectomy	None	Surgical excision	14	NR
Gandla et al., 2018 [8]	M	45	NR	Nephrectomy	Adrenal	Radiotherapy	12	NR
Kishore et al., 2018 [9]	M	54	NR	Palliative therapy	Bone, retromolar, mediastinum, bilateral hilar lymphadenopathy, lung, left adrenal	Chemotherapy, radiotherapy	2	NR
Yang et al., 2019 [2]	F	83	Incidental renal mass	Nephrectomy	Lung, left iliac bone, spine	Surgical excision	180	NR
Krogerus et al., 2020 [20]	M	65	Scalp lesion	Nephrectomy	Lung	Surgical excision	0	NR
Altinkaya et al., 2021 [21]	M	77	Flank pain and fatigue	Chemotherapy	Lung, diaphragmatic pleura, neck lymph nodes, mediastinum, abdomen	Surgical excision	144	5
Leve et al., 2021 [4]	M	75	Incidental renal mass	Nephrectomy	None	Surgical excision	84	NR
Singla et al., 2022 [22]	M	53	Weight loss, flank mass	Nephrectomy, chemotherapy	Upper jaw	Surgical excision, chemotherapy	17	NR
Meshikhes et al., 2023 [1]	M	54	Incidental renal mass	Nephrectomy	Spine	Surgical excision	204	Cardiac arrest
Our case	M	69	Hematuria, scalp metastasis	Planned for systemic therapy	Hilar lymph nodes, adrenal, lung, bone	NR	0	1

The clinical presentation of scalp metastasis typically appears as a firm, well-defined, purple-to-reddish mass. Additional features may include bleeding, a painless vegetative appearance, ulceration, or an expanding pulsatile lesion. Diagnosing RCC cutaneous metastasis early can be challenging, as it may mimic other dermatological conditions, such as melanoma, pyogenic granuloma, cutaneous angiosarcoma, basal cell carcinoma, or hemangioma [24].

Despite significant research on RCC dissemination, the precise mechanisms remain unclear. Proposed pathways include hematogenous metastasis, lymphatic spread via the thoracic duct, and direct invasion or implantation from surgical procedures. Hematogenous spread is considered the primary mechanism [1]. Due to RCC’s highly vascular nature, tumor cells may disseminate through the renal vein to various organs. For instance, lung metastasis occurs when tumor cells travel from the renal vein to the inferior vena cava and reach the right atrium and lungs. The valveless vertebral veins, known as Batson’s plexus, could be a route for RCC cells to spread from the renal vein to the emissary scalp veins, ultimately reaching the scalp and skin [1,2].

Management of RCC typically involves partial or radical nephrectomy, which can also serve as a cytoreductive approach in cases of metastatic RCC [25]. Surgical excision is generally the preferred treatment for scalp metastases [1]. However, when surgery is not feasible, alternative treatments, such as chemotherapy, radiotherapy, or interferon, may be considered [5,7-10]. In our case, the patient was scheduled for systemic therapy, but his condition rapidly deteriorated, and he died four weeks after his initial presentation.

With the advent of targeted therapies, conventional chemotherapy has become less effective for metastatic RCC. Tyrosine kinase inhibitors, such as sorafenib, sunitinib, bevacizumab, pazopanib, and lenvatinib, have shown promise by targeting the vascular endothelial growth factor signaling pathway. Sunitinib and pazopanib are approved as first-line therapies, while mammalian targets of rapamycin inhibitors, including everolimus and temsirolimus, are used as second-line treatments. Cytokines such as interleukin-2 and interferon alfa are also available, though their response rates and progression-free survival are generally lower than those of sunitinib [1].

Skin metastasis from RCC is generally a poor prognostic factor, with up to 90% of cases involving concurrent visceral metastases [26]. The median survival for metastatic clear cell RCC is approximately 13 months, with a five-year survival rate of less than 10% [27,28]. A review of the literature revealed that 11 patients died within an average of 7.6 months after the detection of scalp metastasis [1].

## Conclusions

Metastasis of RCC to the scalp is rare and usually associated with a poor prognosis. Maintaining a high level of suspicion for scalp metastasis is important, particularly when it is the first manifestation, which occurs in approximately one-third of cases. Scalp metastasis is often diagnosed in the later stages of the disease, even following nephrectomy. Surgical excision is the preferred treatment option, though chemotherapy, radiotherapy, or interferon may be considered when surgery is not viable.
